# The utility of all-freeze IVF cycles depends on the composition of study populations

**DOI:** 10.1186/s13048-023-01269-0

**Published:** 2023-09-11

**Authors:** Norbert Gleicher, Sarah K. Darmon, Pasquale Patrizio, David. H. Barad

**Affiliations:** 1 The Center for Human Reproduction, 21 East 69th Street, New York, NY 10021 USA; 2 The Foundation for Reproductive Medicine, New York, NY USA; 3 Stem Cell Biology and Molecular Embryology Laboratory, The Rockefeller University, New York, NY USA; 4 Department of Obstetrics and Gynecology, Medical University of Vienna, Vienna, Austria; 5 Department of Obstetrics, Gynecology and Reproductive Sciences, University of Miami, Miller School of Medicine, Miami, FL USA

**Keywords:** Infertility, In vitro fertilization (IVF), Embryo freezing, Embryo banking, All-freeze cycles, Add-ons to IVF

## Abstract

**Background:**

Because often introduced without proper validation studies, so-called “add-ons” to IVF have adversely affected in vitro fertilization (IVF) outcomes worldwide. All-freeze cycles (embryo banking, EB) with subsequently deferred thaw cycles are such an “add-on” and, because of greatly diverging reported outcomes, have become increasingly controversial. Based on “modeling” with selected patient populations, we in this study investigated whether reported outcome discrepancies may be the consequence of biased patient selection.

**Results:**

In four distinct retrospective case control studies, we modeled in four cohort pairings how cryopreservation with subsequent thaw cycles affects outcomes differently in good-, average- and poor-prognosis patients: (i) 127 fresh vs. 193 frozen donor-recipient cycles to model best-prognosis patients; (ii) 741 autologous fresh non-donor IVF cycles vs. 217 autologous frozen non-donor IVF cycles to model average prognosis patients; (iii) 143 favorably selected autologous non-donor IVF cycles vs. the same 217 frozen autologous cycles non-donor to monitor good- vs. average-prognosis patients; and (iv) 598 average and poor-prognosis autologous non-donor cycles vs. the same 217 frozen autologous non-donor cycles to model poor vs. average prognosis patients. In best-prognosis patients, EB marginally improved IVF outcomes. In unselected patients, EB had no effects. In poor-prognosis patients, EB adversely affected IVF outcomes. Unexpectedly, the study also discovered independent-of-age-associated chromosomal abnormalities, a previously unreported effect of recipient age on miscarriage risk in donor-egg recipients.

**Conclusions:**

In poor-prognosis patients, EB cycles should be considered contraindicated. In intermediate-prognosis patients EB does not appear to change outcomes, not warranting additional cost and time delays. Therefore, only good-prognosis patients are candidates for EB, though they will experience only marginal benefits that may not be cost-effective.

## Background

Traditionally, embryo cryopreservation during in vitro fertilization (IVF) has mostly been a last resort option when fresh embryo transfers were either contraindicated and/or more embryos were produced than could be transferred. Some colleagues recently, however, advanced a concept of all-freeze IVF cycles with routine embryo banking (EB), claiming improved pregnancy and live birth chances [1–3].

Like other “add-ons” to IVF, EB has therefore remained a controversial hypothesis [4–6], as “add-ons” without proper prior validations since 2010 have become more common [7] and declining live birth rates in fresh non-donor IVF cycles have been attributed to their increasing utilization [8]. A recent review summarized why the EB hypothesis may be incorrect [9]. In many “add-ons,” biased patient selections were identified as a major reason. Regarding EB, this can be extrapolated from two large, almost identical, Chinese multi-center studies, executed by the same group of investigators with, nevertheless, opposing outcomes: Comparing fresh transfers to transfers after EB, one study demonstrated mild outcome benefits from EB [10], while the other did not [11]. Both studies were performed on women with identical genetic background (Han Chinese); their only difference was that the former study transferred embryos at blastocyst stage and the latter at cleavage stage.

Blastocyst stage transfers, however, favor the selection of better-prognosis patients since study inclusion mandated at least one blastocyst-stage embryo, a qualification in poorer-prognosis patients often not achieved. Yet, many studies then generalize conclusions in such favorable-prognosis patients to *all *IVF patients. The second Chinese study, which transferred embryos already at cleavage stage, did not bias patient selection in the same way, therefore utilizing a much better representation of a general patient population of infertile women and found no outcome advantage from EB. Unsurprisingly, the marginal outcome advantages seen for EB in the first Chinese study of better-prognosis patients [10], thus, promptly evaporated in the second study that transferred at cleavage-stage [11], perfectly demonstrating the often-overlooked effects of inappropriate patient selection. Appropriately adjusted studies have confirmed this [1, 3, 10–12] utilizing genetically, distinctively different patient populations from Han Chinese, including a recent European multicenter study also performed in favorably selected patients receiving single blastocyst-stage embryo transfers [12].

General infertile patient populations always contain a mix of good-, average-, and poor-prognosis individuals [13]. Correct considerations of study populations are therefore essential for the correct interpretation of study results. To confirm that contradictory outcome data following EB may be the consequence of biased patient selections, here presented study was conceived, using on purpose highly selected patient populations in a retrospective analysis of a large data set of IVF cycles to model outcome comparisons for IVF with and without the two quintessential components of EB,—cryopreservation and thaw-cycles. As our data will demonstrate, our models produced basically identical results to recently reported studies that demonstrated no significant outcome benefits for EB [12].

## Results

### Third-party egg donation cycles: fresh vs. frozen

In this first part of the study, we compared IVF cycle outcomes in 127 fresh donor egg recipient cycles to 193 frozen-thawed cycles in which embryos had been produced with fresh donor eggs (Table 1).

**Table 1:** Comparison of fresh and frozen donor egg recipient cycles

	Fresh Cycles	Frozen Cycles	*P*-value/adjusted
Number of cycles	127	193	
Age (years)	45.6 ± 5.1	45.7 ± 5.9	0.9330
AMH (ng/mL)	0.4 ± 0.5	0.6 ± 0.8	0.0775
Highest FSH (mIU/mL)	27.5 ± 31.0	24.5 ± 26.9	0.4213
Number embryos transferred	1.6 ± 0.6	1.7 ± 0.6	0.2208
Pregnancies (n/%)	46 (36.2%)	56 (29.0%)	0.1760/0.2487
Live births (n/%)	34 (73.9%)	42 (75.0%)	0.9003/0.1735
Miscarriages (n/%)	12 (26.1%)	14 (25.0%)	0.9003/0.1735

#### Patient characteristics

As the table demonstrates, the ages of recipient patients were very advanced and similar (45.6 ± 5.1 and 45.7 ± 5.9 years; *P* = 0.9330), as were lowest Anti-Müllerian hormone (AMH) (0.4 ± 0.5 vs. 0.6 ± 0.8 ng/mL; *P* = 0.0775) and highest Follicle-stimulating hormone (FSH) levels (27.5 ± 31.0 vs. 24.5 ± 26.9; *P* = 0.4213) as well as numbers of transferred embryos (1.6 ± 0.6 vs. 1.7 ± 0.6; *P* = 0.2208).

#### IVF outcomes

In fresh donor-recipient cycles, 46/127 (36.2%) conceived and 34/46 (73.9%) delivered, while 12/46 (26.1%) miscarried. In contrast, 56/193 (29.0%) frozen cycles led to clinical pregnancy (*P* = 0.1760), 42/56 (75.0%) delivered, and 14/56 miscarried (25.0%). Adjustments for age and AMH values, barely moved the *P*-value (*P* = 0.2487), reaffirming the close match of both study groups.

### Autologous non-donor cycles: fresh vs. frozen

Here, we had 741 fresh transfer cycles and 217 frozen cycles for comparison, with embryo numbers transferred again being similar, but patient characteristics significantly differing (Table 2).

**Table 2:** Comparison of autologous fresh non-donor to frozen autologous non-donor cycles

	Fresh Cycles	Frozen Cycles	*P*-value/adjusted
Number of cycles	741	217	
Age (years)	41.2 ± 4.8	39.5 ± 5.9	< 0.0001
AMH (ng/mL)	1.2 ± 1.7	2.4 ± 2.6	< 0.0001
Highest FSH (mIU/mL)	14.8 ± 12.7	11.5 ± 7.8	0.0003
Number embryos transferred	2.2 ± 1.2	2.1 ± 1.0	0.2536
Pregnancies (n/%)	82 (11.1%)	42 (19.4%)	0.0014/0.2991
Live births (n/%)	53 (64.3%)	26 (61.9%)	0.7648/0.5189
Miscarriages (n/%)	29 (35.4%)	16 (28.1%)	0.7648/0.5189

#### Patient characteristics

Patients who had fresh cycles were significantly older (41.2 ± 4.8 vs. 39.5 ± 5.9 years (*P* < 0.0001) and had significantly lower AMH (1.2 ± 1.7.vs. 2.4 ± 2.6 ng/mL; *P* < 0.0001) and significantly higher FSH (14.8 ± 12.7 vs. 11.5 ± 7.8; *P* = 0.0003) Embryo numbers transferred were, however, similar: 2.2 ± 1.2 vs. 2.1 ± 1.0 (*P* = 0.2536).

#### IVF outcomes

Fresh cycles in this head-to-head comparison demonstrated significantly lower clinical pregnancy rates (82/741; 11.1%) than frozen cycles (42/217; 19.4%; *P* = 0.0014). Yet, once outcomes were adjusted for age and AMH, the significant outcome difference between both groups became statistically insignificant (*P* = 0.2991). Of the pregnancies in frozen cycles, 26/42 (61.9%) delivered, while 16/42 (38.1%) miscarried. While in fresh cycles 53/82 (64.6%) delivered and 29/82 miscarried (35.4%), (*P* = 0.7648).

### Autologous non-donor cycles: fresh vs. frozen cycles in favorably selected patients

Here, the frozen group remained the same as in the preceding comparison, but fresh cycles were selected for favorable patients by selecting cycles of 143 women who produced enough embryos in fresh cycles to have at least one embryo cryopreserved. That this group represented only 19.0% of all 741 fresh cycles, again reflects the overall poor prognosis of this patient cohort (Table 3). This left 598 cycles, now presumably representing women with average and poor prognoses (Table 4).

**Table 3:** Comparison of best-prognosis autologous fresh non-donor to frozen autologous non-donor cycles

	Fresh Cycles	Frozen Cycles	*P*-value/adjusted
Number of cycles	143	217	
Age (years)	37.1 ± 4.9	39.5 ± 5.9	0.0001
AMH (ng/mL)	2.4 ± 2.2	2.4 ± 2.6	0.8457
Highest FSH (mIU/mL)	10.9 ± 11.2	11.5 ± 7.8	0.5717
Number embryos transferred	2.3 ± 1.0	2.1 ± 1.0	0.0874
Pregnancies (n/%)	45 (31.5%)	42 (19.4%)	0.0086/0.0451
Miscarriages (n/%)	14 (31.1%)	16 (28.1%)	0.4934/0.4693

**Table 4:** Comparison of intermediate and poor-prognosis autologous fresh non-donor cycles to frozen autologous non-donor cycles

	Fresh Cycles	Frozen Cycles	*P*-value/adjusted
Number of cycles	598	217	
Age (years)	42.2 ± 4.2	39.5 ± 5.9	< 0.0001
AMH (ng/mL)	0.9 ± 1.4	2.4 ± 2.6	< 0.0001
Highest FSH (mIU/mL)	15.8 ± 12.9	11.5 ± 7.8	< 0.0001
Number embryos transferred	2.2 ± 1.2	2.1 ± 1.0	0.3874
Pregnancies (n/%)	37 (6.2%)	42 (19.4%)	< 0.0001/0.0028
Live births (n/%)	22 (59.5%)	26 (61.9%)	0.8242/0.4921
Miscarriages (n/%)	15 (40.5%)	16 (28.1%)	0.8242/0.4921

#### Patient characteristics

In this analysis, demographics of favorably selected fresh cycle patients significantly differed from the preceding analysis: While in the whole group of 741 women, women with fresh had been significantly older than patients undergoing frozen-thawed cycles (Table 2), the now favorably selected women among that group of patients were significantly younger (37.1 ± 4.9 vs. 39.5 ± 5.9 years; *P* = 0.0001) than women having frozen-thawed embryo transfers. Moreover, neither AMH (2.4 ± 2.2 vs. 2.4 ± 2.6 ng/mL; *P* = 0.8457) nor FSH (10.9 ± 11.2 vs. 11.5 ± 7.8 mIU/mL; *P* = 0.5717) were any longer significantly different. In addition, a non-significant trend toward larger embryo transfer numbers developed in fresh cycles (2.3 ± 1.0 vs. 2.1 ± 1.0; *P* = 0.0874).

Comparing the remaining 598 average and poor-prognosis patients between fresh and frozen transfers, patients who had fresh cycles were significantly older (42.2 ± 4.2 vs. 39.5 ± 5.9 years (*P* < 0.0001), had significantly lower AMH (0.9 ± 1.4.vs. 2.4 ± 2.6 ng/mL; *P* < 0.0001) and significantly higher FSH (15.8 ± 12.9 vs. 11.5 ± 7.8; *P* < 0.0001). Embryo numbers transferred were, however, again similar: 2.2 ± 1.2 vs. 2.1 ± 1.0 (*P* = 0.3874, Table 4).

#### IVF outcomes

In this scenario, however, 45/143 clinical pregnancies occurred in fresh cycles (31.5%) vs. only 42/217 (19.4%) in frozen cycles, producing a significant difference to the benefit of fresh cycles (*P* = 0.0086), which remained significant upon adjustment for age and AMH (*P* = 0.0451). Of the pregnancies in the frozen cycle, 26/42 (61.9%) delivered, while 16/42 (38.1%) miscarried. While in fresh cycles, 31/45 (68.9%) delivered and 14/45 miscarried (31.1%), (*P* = 0.4934).

In contrast, the remaining average- and poorer-prognosis patients, 37/598 (6.2%) had clinical pregnancies with 22 (59.5%) live births and 15 (40.5%) miscarriages. This was an insignificant finding when compared to frozen cycles (*P* = 0.8242, Table 4).

## Discussion

Due to our center’s very adversely selected patient population, widely applied infertility treatments are often not applicable. For example, closed incubation and imaging systems found in our patients produce similar outcomes to standard embryology in third-party egg donor cycles, but adversely affect IVF outcomes in poor-prognosis patients still pursuing treatments with autologous oocytes [14]. EB attracted skepticism for several reasons: In-house data questioned the hypothesis that ovarian hyperstimulation adversely affects embryo implantation [6, 15]. Especially in poorer-prognosis patients we also were concerned about the adverse effects of cryopreservation on cumulative pregnancy chances. We also questioned the additional costs of thaw cycles and, of course, noted that reported improved pregnancy and live birth rates after EB occurred only in good-prognosis patients [1–3, 10, 12]. Furthermore, with good-prognosis patients to minor degrees, benefit from EB since unselected patient populations demonstrate no consequences from EB, they must contain a counterbalancing group of patients who experience detrimental effects, such as older or younger women with low functional ovarian reserve (LFOR).

Here presented data confirmed this, leading to the following conclusions (i) In unselected patient populations EB does not improve IVF outcomes. (ii) In favorably selected patients, EB with reference embryo transfer minimally improves pregnancy and live birth chances. (iii) EB in poorer-prognosis patients, however, exert compensatory detrimental effects. (iv) All of these observations, as here demonstrated in a four-step study, also apply in poor-prognosis patients, and are here detailed:

### Best prognosis patients: third-party egg donation cycles; fresh vs. frozen

A careful case-controlled study of practically identical patient groups in fresh and frozen third-party donor egg cycles (i.e., best-prognosis patients, Table 1), demonstrated no difference in clinical pregnancy rates (*P* = 0.1760) even after adjustments for age and AMH (*P*= 0.2487). This study can be viewed as a baseline control model, demonstrating no visible effect of embryo freezing on IVF outcomes in unselected patients and reaffirms prior prospectively randomized studies of unselected patient populations [11].

One unexpected observation, however, deserves further comments: In Table 1 reported almost identical miscarriage rates in donor-recipient cycles fresh (26.1%) or frozen thawed (25.0%), which are unexpectedly high for young third-party egg donors. The 2016 CDC National ART Summary Report suggested in third-party egg donation cycles only an approximately 10.4% miscarriage rate (https://www.cdc.gov/art/pdf/2016-report/ART-2016-National-Summary-Report-pdf), suggesting that here observed more than double as high rate in both study groups must reflect the very advanced (also practically identical) ages of both recipient patient groups (45.6 ± 5.1 and 45.7 ± 5.9 years, respectively). Surprisingly, the literature does not inform on miscarriage rates depending on recipient age. Two publications commented peripherally on the subject, with one noting no differences [16] and the other noting small increases in pregnancy losses [17]. Here reported outcome data, therefore, for the first time offer strong evidence that recipient age increases miscarriage risk. The likely reasons are accumulating medical problems, unrelated to the age of oocytes and, therefore, unrelated to chromosomal abnormalities.

### Autologous non-donor cycles: fresh vs. frozen

In part two of here presented study, we switched to the investigation of the use of autologous oocytes in an, overall, highly unfavorable patient population and, as a first step, simply compared all fresh and all frozen-thawed cycles (Table 2). In contrast to previously described third-party donor cycles, demonstrated two highly divergent patient populations; Fresh cycles, not only were three times as common but also represented significantly older women (*P* < 0001), with much lower AMH (*P* < 0.0001) higher FSH (*P* = 0.0003), though a very similar number of transferred embryos. Women who underwent frozen-thawed cycles were not only significantly younger, but also had a much better functional ovarian reserve. That they achieved significantly better clinical pregnancy rates (*P* = 0.0014) with the transfer of identical embryo numbers (*P* = 0.2536), therefore, cannot surprise and does *not* suggest that this improved outcome is the consequence of delayed frozen-thawed embryo transfers.

This is confirmed by adjustment for age and AMH (as a representative of LFOR) making the significant difference in pregnancy rate disappear (*P* = 0.2991). Adjusting for FSH instead of AMH, made no difference (*P* = 0.1564). Since both reflect LFOR, we formally adjusted for only one (AMH). Seeing improvements in IVF cycle outcomes in frozen-thawed over fresh cycles with the use of autologous eggs, under those circumstances, therefore, are mostly due to underlying patient characteristics and not caused by EB.

Though both patient groups in this study section are significantly younger than in the above-presented third-party-donor section, fresh and frozen cycles, still, involved older women (41.2 ± 4.8 vs. 39.5 ± 5.9 years, respectively). Frozen cycles were, however, performed in younger women than fresh cycles (*P* < 0.0001). That miscarriages were nominally lower in frozen cycles (28.1%) than fresh cycles (35.4%) here, therefore, has no practical meaning. That miscarriages were uniformly higher in autologous than donor-recipient cycles, whether fresh or frozen, even though donor egg recipients were significantly older, is, however, of interest. Third-party egg donation in older women, therefore, clearly does reduce miscarriage risk in comparison to the use of autologous oocytes. Likely due to non-chromosomal maternal causes this advantage, however, shrinks with advancing recipient age.

### Autologous non-donor cycles: fresh vs. frozen cycles in good-, intermediate- and poor-prognosis patients

Addressing the third and fourth steps of this study (Tables 3 and 4), we argued that every study population, including our 741 fresh cycles, can be divided into better- average- and poorer-prognosis patients [13]. Since, after female age, transferrable embryo numbers are the second-most important predictor of pregnancy chances in IVF [13], numbers of embryos produced in a cycle allow the identification of a best-prognosis sub-group. Further patient selection can be achieved by selecting patients who produced more embryos than immediately transferrable (i.e., achieved cryopreservation). Consequently, 143 (19%) women among 741 ended up qualified as best-prognosis patients. In the third study, they now were in fresh transfers compared to the 217 frozen embryo transfer cycles from the earlier analysis.

Changing the patient selection, of course, resulted in highly significant outcome changes: Women undergoing fresh cycles now were, suddenly, significantly younger (*P* = 0.0001) (previously significantly older) in the complete autologous group (*P* < 0.0001) and significant differences in AMH and FSH to the benefit of frozen cycles completely disappeared, together with all prior outcome advantages in pregnancy rates for frozen cycles (*P* = 0.0086 before and *P* = 0.0451 after adjustment for age and AMH).

Comparing then the remaining 598 moderate and poor-prognosis fresh cycles with the 217 frozen cycles, patient demographics again reverted into similar ranges as had been seen previously in Table 2 for the complete autologous patient populations, with frozen transfer cycles seemingly outperforming pregnancy rates in fresh cycles. (*P* < 0.0001, *P* = 0.0028, Table 4).

## Limitations and conclusions

The principal limitation of this study is its retrospective nature. Considering the homogeneity of here utilized patient populations and statistical adjustments, this here presented study format, however, offers information even some well-designed prospectively randomized studies cannot provide. A 2018 Society for Assisted Reproductive Technology (SART) study reached similar conclusions, suggesting that universal embryo freezing only serves good responders [5]. Since the completion of this study, another retrospective study in a general infertile population was published. Once again, the study demonstrated outcome benefits for an all-freeze strategy in only good-prognosis patients, this time defined as women below age 35 [18].

In poor-prognosis patients that were never before studied within this framework, every single oocyte and embryo is of much greater importance than in women with average and good prognoses. Therefore, they often are the “canary in the mine” in sounding the alarm about otherwise, ineffective treatments. Considering that ineffective treatments increase costs and, in this case, also result in delays in treatments, this study offers solid new evidence that the concept of universal all-freeze cycles with subsequently delayed frozen-thawed cycles must be reconsidered. Under the best of all circumstances, it should only be restricted to best-prognosis patients. Similar population dynamics are, likely, also relevant in association with other recent “add-ons” to IVF [8].

## Methods

### Participants

Our center, likely, serves the prognostically most unfavorable patient population among all reporting IVF centers in the U.S. We serve the oldest patient population with a median age of over 43 years between 2016–2019 (national median ~ 36 years). (https://www.cdc.gov/art/artdata/index.html). Over 90% of newly presenting patients have previously failed IVF cycles elsewhere, often at multiple centers, and over 50% have been advised that third-party egg donation is their only remaining chance of pregnancy. Our center also treats disproportionally large numbers of phenotype-D polycystic ovarian syndrome (PCOS) patients, after age 35 more treatment-resistant than other PCOS phenotypes to standard fertility treatments, including IVF [19]. Even younger patients, almost without exception, also previously failed IVF cycles and presented with abnormal LFOR. Yet, as we previously demonstrated, even such an overwhelmingly unfavorable patient population is, still, made up of relatively good-, intermediate-, and poor-prognosis patients [13]. Our center’s patient population, therefore, appears well-suited for here presented retrospective case control studies between carefully selected sub-populations. Taking advantage of these circumstances, here presented study investigated between 2017 – 2020, in our center’s patient population the effects of embryo freezing, followed by thaw-cycles in infertile women in four distinct patient groupings:

- (i) Here we compared IVF cycle outcomes in 127 infertile women who had fresh embryos transferred, produced from young anonymous egg donors and, therefore, represented best-prognosis patients, to 193 infertile women who had frozen-thawed embryos transferred, produced with oocytes from young donors (Table 1). Due to the very advanced ages in both recipient groups mandating avoidance of multiple pregnancies, almost all transfers were elective single embryo transfers. This model, thus, reflected EB in best-prognosis patients.
- (ii) Here we compared outcomes in 741 fresh and 217 frozen embryo transfers, produced with autologous oocytes of infertility patients (Table 2) and, because of younger ages, numbers of transferred embryos were less restricted; small egg and embryo yields due to LFOR, however, still limited transferred embryos to mostly two. This model, thus, evaluated EB in average-prognosis infertility.
- (iii & iv) Here we selected a favorable-prognosis group of 143 women from among above 741 unselected women and compared those two groups (Table 3). In addition, we also compared the remaining 598 unselected women to the same group of 217 frozen autologous IVF cycles, also used in the second investigation (Table 4). The selected sub-group of 143 prognostically favorable patients was defined by their ability to produce larger embryo yields in fresh IVF cycles, allowing for cryopreservation of extra-numeral embryos. This selection criterion was based on the reported observation that, even in poor-prognosis patients, after female age, the number of transferrable embryos in an IVF cycle represents the second-most important predictor of IVF success [13].

All patients were consecutively entered into the center’s anonymized electronic research data bank during the study years after providing informed consent. Excluded were repeat cycles and cycles that were cancelled before embryo transfer. The so-excluded patients obviously eliminated worst-prognosis patients. Here reported observations in poor-prognosis patients may, therefore, be mild underestimations.

### IVF cycles

Oocyte donation cycles of anonymous egg donors were stimulated in long agonist protocols, using a human menopausal gonadotropin (hMG) product at a dosage of 225 IU daily.

Autologous cycles were only initiated after prior priming of ovaries if patients were over age 40 years and/or demonstrated low functional ovarian reserve (LFOR), defined as abnormally high age-specific FSH [20] and/or abnormally low age-specific AMH [21]. Priming involved supplementation with dehydroepiandrosterone (DHEA; Fertinatal®, Fertility Nutraceuticals, LLC, New York, N.Y), 25 mg TID, for at least six weeks or until androgen and sex hormone binding globulin (SHBG) were in normal ranges. All patients, in addition, received the antioxidant CoQ10 at a dosage of 900-1000 mg/day (OvoEnergen®, Fertility Nutraceuticals, LLC, New York, N.Y.). A large majority of autologous cycles received direct gonadotropin stimulation, starting on day-2 of menses with daily gonadotropins (300–450 IU of an FSH product and 150 IU of an hMG product, both from different manufacturers based on patient preference and/or insurance mandates). Due to patients having undergone highly individualized egg retrieval (HIER) [22, 23] and, therefore, had early egg retrievals, they did not require either agonists or antagonists to prevent premature ovulation. The default method in some younger patients who were not expected to have early retrievals was a previously described microdose agonist protocol first reported by Surrey et al. [24]. Ovulation was triggered with 10,000 IU of human chorionic gonadotropin (hCG, from different manufacturers).

Except for very rare exceptions in donor-recipient cycles, embryo transfers occurred at cleavage-stage (usually day-3; but sometimes day-2). Similarly, except for rare exceptions, embryos did not undergo preimplantation genetic testing for aneuploidy (PGT-A).

A clinical diagnosis of pregnancy required visualization of at least one gestational sac with a fetal heart on ultrasound. A diagnosis of clinical miscarriage required at least prior visualization of a gestational sac. Chemical pregnancies were not considered in here reported statistics.

### Statistical analyses

Patient demographics were compared by a two-sample t-test and presented as mean and standard deviation. Clinical pregnancies, live birth, and miscarriage rates were compared with a Chi-square test and logistic regression model, controlling for age and AMH. All statistics were performed using SAS version 9.4. A *P*-value < 0.05 was considered statistically significant.

### Institutional Review Board (IRB)

This study was approved by the center’s IRB on an expedited basis since here reported data were extracted from the center’s anonymized electronic patient database, which includes all data from patients who signed a written consent that allowed the use of their medical record data for research purposes, as long as those data remained confidential, and their identity remained protected.

## Data Availability

All data generated or analyzed during this study are included in this published article. The datasets used and/or analyzed during the current study are available from the corresponding author upon reasonable request.

## Abbreviations

IVF: In vitro fertilization
EB: Embryo banking
AMH: Anti-Müllerian hormone
FSH: Follicle-stimulating hormone
LFOR: Low functional ovarian reserve
PCOS: Polycystic ovarian syndrome
DHEA: Dehydroepiandrosterone
SHBG: Sex hormone binding globulin
HIER: Highly individualized egg retrieval
hCG: Human chorionic gonadotropin
hMG: Human menopausal gonadotropin
SHBG: Sex hormone binding globulin
IRB: Institutional Review Board
